# An assessment of hospital maternal health services in northern Ghana: a cross-sectional survey

**DOI:** 10.1186/s12913-020-05937-5

**Published:** 2020-11-26

**Authors:** Edward Kwabena Ameyaw, Roberta Mensima Amoah, Carolyne Njue, Nguyen Toan Tran, Angela Dawson

**Affiliations:** 1 Australian Centre for Public and Population Health Research, University of Technology Sydney, Sydney, Australia; 2 Department of Public Health, School of Allied Sciences, University for Development Studies, Tamale, Northern Region Ghana

**Keywords:** Maternal healthcare, Obstetric care, Reproductive health, Referral, Northern Ghana

## Abstract

**Background:**

Access to and delivery of comprehensive emergency obstetric and neonatal care (CEmONC) services are often weak in low and middle-income countries affecting maternal and infant health outcomes. There are no studies on resources for maternal healthcare in the Northern region of Ghana. This knowledge is vital for health service planning and mobilising funding to address identified gaps. We investigated the available resources for managing CEmONC and referral services in the region.

**Methods:**

This study involved a cross-sectional survey of maternity facilities in ten hospitals in the Northern region of Ghana, serving a population of 2,479,461, including 582,897 women aged 15–49. Public and faith-based hospitals were included in the study. We used the Service Provision Assessment tool to gather data for this study between October and December 2019. Given the small sample size, we used descriptive statistics to summarise the data using SPSS version 25 and Excel 2016.

**Results:**

A total of 22,271 ANC visits from women to these hospitals occurred in the past 3 months preceding the study; however, 6072 birth events (cases) occurred within the same period. All the hospitals had less than one general medical doctor per 10,000 population (range 0.02–0.30). The number of midwives per 10,000 population ranged from 0.00 (facility H and J) to 1.87 (facility E), and none of the hospitals had a university-trained nurse designated for maternity care. Only one hospital had complete equipment for emergency obstetric and newborn care, while four others had adequate emergency obstetric care equipment. The number of maternity and delivery beds per 10,000 population was low, ranging from 0.40 to 2.13.

**Conclusions:**

The management of emergency obstetric care and referrals are likely to be affected by the limited human resources and equipment in hospitals in Northern Ghana. Financial and non-financial incentives to entice midwives, obstetricians and medical officers to the Northern region should be implemented. Resources should be mobilised to improve the availability of essential equipment such as vacuum extractors and reliable ambulances to enhance referral services. Considerable health system strengthening efforts are required to achieve the required standards.

## Background

A successful health system is the one that links people to the required level of care so that they receive prompt care in a timely fashion [1]. Research examining referral systems in low and middle-income countries has found that the quality of and access to emergency healthcare are influenced by geography, the distribution, and location of health facilities, communication, socio-demographic and cultural dynamics [2–4]. Access to Basic and Comprehensive Emergency Obstetric and Neonatal Care (BEmONC, CEmONC) is an essential strategy for averting maternal and neonatal mortality [4] and is reliant on a well-coordinated referral system [5, 6]. The availability and quality of EmONC services in low and middle-income countries are limited and have been attributed to problems with referral systems [7–9]. Service Provision Assessments (SPA) across low and middle income countries have identified critical gaps. For example, an assessment of health facilities in Tanzania revealed that they were less likely to have an emergency transportation system (10%) [10]. In Kenya, an SPA found that, health facilities generally performed well, however, private facilities were more accessible [11].

The third Sustainable Development Goal (SDG) urges all countries to institute measures that can reduce maternal deaths below a maternal mortality ratio (MMR) of 70 per 100,000 live births by 2030. This SDG calls on countries to reduce newborn deaths to 12 deaths per 1000 live births within the same period [12]. As of 2017, Ghana’s MMR was 310 deaths per 100,000 while neonatal mortality rate (NMR) stood at 25 per 1000 live births [13].

More concerted efforts are required to achieve high-quality maternal healthcare and ensure better inter-facility linkages. Essential interventions or signal functions are needed to treat serious obstetric complications that contribute to reducing maternal mortality and preventing adverse birth outcomes [14]. The availability of the signal functions corresponds to the level of emergency obstetric and newborn care of facilities where these are provided [14]. At the first level of care, BEmONC comprises parenteral antibiotic administration, uterotonic drugs administration, administration of parenteral anticonvulsants for preeclampsia and eclampsia, manual removal of placenta, removal of retained products, performing assisted vaginal delivery, and performing basic neonatal resuscitation; at the referral level of care, CEmONC includes performing caesarean-section surgery and blood transfusion in addition to the seven BEmONC interventions [14]. Furthermore, WHO recommends a ratio of 2.28 midwives/doctors per 1000 population [15, 16]. Therefore, maternal complications can be effectively managed if health facilities are equipped and staffed optimally [17].

Since independence in 1957, the Northern region of Ghana has experienced an inequitable distribution of health workers and other essential resources [18]. The harsh climatic and high levels of poverty in the region contribute to its disadvantaged status [19]. The southern region of Ghana in comparison, has a more highly developed infrastructure and accommodates the head offices of central Government ministries and businesses. Between 1991 and 2006 poverty declined in the south by 58.7%, while in the northern region only experienced a 8.9% reduction in poverty during this period [18].

The Northern region, has 10.2% of the nation’s population, but only 2% (38) of the country’s medical officers and 7.4% (285) of midwives. The southern region of Volta in comparison has 8.6% of Ghana’s population while it has 2.7% (43) of the nation’s medical officers and 10.1% (386) of all midwives. Similar trends exist in the distribution of health staff in other categories [20]. Successive governments have tried to incentivize the health workforce to accept postings to the Northern region. Among these measures are the Deprived Area Incentive Scheme, where a 20–35% allowance and basic salary are provided tor those who accept a posting to the region coupled with housing [21].

However, these incentives have not resulted in a change in workforce distributions. In 2016, the Greater Accra and Northern region had doctor-patient ratios of 1:3136 and 1:18,380, respectively [22]. An ambulance audit in 2018 also revealed that the Northern region had six of the 55 functional ambulances available for use in the country [23]. In contrast, the Central region had nine of these ambulances to serve 9.2% of the nation’s population. This has necessitated the use of donkeys and tricycles for some maternal referrals in the Northern region [24]. The absence of transport has been linked to an increase in perinatal death [25] and home delivery [26] in several African countries.

The lack of transport can significantly compromise the ability of CEmONC level health facilities to function optimally. Studies of Emergency Obstetric Care (EmOC) services in the Upper East region of Ghana found limited availability of transport, equipment and supplies for maternity care [27]. A range of factors such as inadequate medical supplies, drugs, poor referral processes and a lack of reliable communication was found to compromise women’s access to essential maternal health services [27, 28]. There are no studies that examine the available resources for maternal healthcare in the Northern region of Ghana and the management of maternal referrals within the region. Therefore, we sought to investigate the availability of resources for managing maternity conditions and how maternal referral services are handled and delivered in this region. These findings provide a useful baseline for decision-makers regarding the resources required to improve maternal health care.

### Theoretical framework

This study is underpinned by functionalist theory [29, 30] that argues that the ability of social institutions, such as health facilities, to address human needs justifies their existence. Each institution influences the form, shape and sustainability of society by providing their core duties. Consequently, the institutions’ inability to offer such roles would render them obsolete [29, 30]. The terms ‘functional’ and ‘dysfunctional’ describe the impact of institutions on society. For instance, a hospital is deemed functional if it has all the necessary resources and executes its role accordingly, contrarily, it is dysfunctional if it operates below its required mandate [29, 30].

It is noteworthy that a health facility can be functional and dysfunctional simultaneously. For hospitals to provide optimum maternity healthcare and well streamlined maternal referral, the health facility must be functional. A functional healthcare provider is a provider who identifies signal function(s), act professionally, and refer cases in line with recommended guidelines.

## Methods

### Study design

This study used a cross-sectional survey in ten hospitals in the Northern region of Ghana to assess the available resources for maternity healthcare including CEmONC and emergency referral. The hospitals comprised all the nine district hospitals in the region; consisting of public (2) and faith-based private-public (7) district hospitals as well as the regional hospital (1). This is the definition of the Ghana Health Service and WHO [22, 31, 32].

### Definitions

Maternity beds refer to all beds available for all maternity or labour related conditions including beds for resting while in labour, beds for childbirth and those used during the immediate postpartum period prior to discharge. On the other hand, delivery beds refer to beds that are exclusively designated for actual childbirth alone.

### Study setting

The Northern region of Ghana is divided into the district, municipal or metropolitan local government areas. The metropolitan area has approximately 250,000 people; a municipality has at least 95,000 people while a district has approximately 75,000 people [33]. For the purpose of this study, the “district” denotes all the three local government areas. There are nine district hospitals across the 16 districts of the region. This study involved all the nine district hospitals and the regional hospital.

### Data collection instrument

We used the Service Provision Assessment (SPA) tool to gather data for this study. The tool has been uploaded as Additional File 1. The SPA was developed by the Demographic and Health Survey (DHS) Program for assessing health facilities in developing countries [34]. The assessment is carried out with a questionnaire that includes questions on facility level infrastructure, resources and systems, maternal and child health services, family planning and sexually transmitted infections [34]. The tool is suitable for assessing maternal and newborn healthcare, child healthcare, infrastructure, resources, systems, and family planning service provision. In 2002, the SPA was used to assess health service delivery in Ghana. It has also been used extensively in other Sub-Saharan Africa countries such as the Democratic Republic of Congo, Egypt, Kenya and Malawi [34]. For this study, we revised the tool to fit the characteristics of the northern region’s health facilities. For instance, the tool aggregates responses when assessing the number of equipment, such as beds. However, most of the facilities assessed had few beds (e.g. 70% had less than five delivery beds), so we reported the actual beds per facility instead of categorising or grouping the number of beds.

### Data collection

Prior to data collection, invitation letters explaining the study’s purpose were sent from the Northern regional health directorate to Medical Superintendents of the nine district hospitals and the regional hospital selected. All heads of the hospital maternal health services were pre-informed by the Medical Superintendents. Following introductions and explanation of the study, we followed up to seek their interest and availability, obtain written informed consent, and schedule appointments with them. We interviewed the heads of maternity health services of the hospitals; however, their assistants were interviewed when it was not possible to meet them in person. Reported figures (e.g. number of ANC visits and number of births) were verified from the records of each facility to ensure accuracy.

All interviews took place at the hospitals during regular working hours. Midwives, all medical officers and midwives in Ghana obtain formal training on EmONC (e.g. caesarean section, vacuum aspiration, blood transfusion, assisted deliveries) as part of their formal training and are also guided by the Safe Motherhood Protocol of Ghana Health Service which dictates how to handle maternity conditions [35]. Data collection was undertaken by two authors (EKA and RMA). EKA is a social scientist public health researcher with much experience in survey administration. RMA is a clinical midwife with expertise in assessing maternity healthcare who clarified the terminology and fieldwork procedures. Interviews lasted between 25 and 40 min. Data collection took place between October 2019 and February 2020.

### Data analysis

Our unit of analysis was at the facility level. We used SPSS version 25 and Excel 2016 for analyses. Given the minimal sample size, we used descriptive statistics to summarise the various aspects of the data. The results are presented in frequencies and percentages. In order to make the findings comparable to global standards as recommended by the WHO [15, 36], in each district/region, we computed the total number of births and ANC attendance within the 3 months preceding the survey per 100,000 (Table 1), maternal healthcare staff per 10,000 (Table 2), and the number of available beds (maternity and delivery beds) per 10,000 (Table 3). Finally, we summarised the available signal functions and essential equipment for maternity healthcare (Table 4).

**Table 1:** Births and ANC attendance in hospitals providing comprehensive emergency obstetric and newborn care per 100,000 population

Facility	Population per district/region	ANC attendance in the past 3 months		Births in the past 3 months	
		n	Per 100,000	N	Per 100,000
A	2,479,461*	7184	289.74	1116	45.01
B	371,351*	6900	1858.08	1400	377.00
C	199,592	1423	712.95	735	368.25
D	123,854*	946	763.80	205	165.52
E	139,283*	467	335.29	848	608.83
F	112,331	499	444.22	342	304.46
G	141,584	1968	1389.99	642	453.44
H	108,816	1116	1025.58	198	181.96
I	65,706*	1020	1552.37	161	245.03
J	111,259	748	672.31	425	381.99
Total	3,853,237	22,271	577.98	6072	157.58

*Population data provided from the 2010 Census report (Ghana Statistical Service [GSS], 2010)

* Further demarcation of regional and district boundaries was done between 2008 and 2019, adding two new regions from the original Northern region and ten new districts within the original Northern region. However, these newly created districts/regions do not have district/regional hospitals, and the population continues to be served by the existing health facilities

**Table 2:** Maternal healthcare staff at the hospitals per 10,000 population

Facility	Population per district/region	General Medical Doctors		Specialist		Non-Physician Clinicians		Anaesthetists		Nurses		University trained nurses		Midwives		Degree midwives		Enrolled Nurses		Allied Professionals	
		N	Per 10,000	n	Per 10,000	n	Per 10,000	N	Per 10,000	n	Per 10,000	n	Per 10,000	N	Per 10,000	n	Per 10,000	n	Per 10,000	n	Per 10,000
A	2,479,461*	4	0.02	0	0.00	0	0.00	4	0.02	1	0.00	0	0.00	15	0.06	4	0.02	0	0.00	0	0.00
B	371,351*	5	0.13	0	0.00	10	0.27	4	0.11	1	0.03	0	0.00	67	1.80	4	0.11	11	0.30	4	0.11
C	199,592	5	0.25	0	0.00	0	0.00	3	0.15	3	0.15	0	0.00	6	0.30	0	0.00	3	0.15	0	0.00
D	123,854*	1	0.08	0	0.00	3	0.24	2	0.16	0	0.00	0	0.00	12	0.97	1	0.08	0	0.00	0	0.00
E	139,283*	2	0.14	0	0.00	0	0.00	2	0.14	0	0.00	0	0.00	26	1.87	1	0.07	4	0.29	0	0.00
F	112,331	3	0.27	0	0.00	0	0.00	2	0.18	0	0.00	0	0.00	8	0.71	1	0.09	3	0.27	0	0.00
G	141,584	1	0.07	0	0.00	0	0.00	1	0.07	3	0.21	0	0.00	8	0.57	0	0.00	5	0.35	0	0.00
H	108,816	1	0.09	0	0.00	2	0.18	2	0.18	0	0.00	0	0.00	0	0.00	6	0.55	2	0.18	0	0.00
I	65,706*	2	0.30	0	0.00	0	0.00	0	0.00	3	0.46	0	0.00	7	1.07	2	0.30	3	0.46	0	0.00
J	111,259	2	0.18	0	0.00	0	0.00	2	0.18	3	0.27	0	0.00	0	0.00	0	0.00	0	0.00	0	0.00
**Total**	3,853,237	26	0.07	0	0.00	15	0.04	22	0.06	14	0.04	0	0.00	149	0.39	19	0.05	31	0.08	4	0.01

*Population data provided from the 2010 Census report (Ghana Statistical Service [GSS], 2010)

* Further demarcation of regional and district boundaries was done between 2008 and 2019, adding two new regions from the original Northern region and ten new districts within the original Northern region. However, these newly created districts/regions do not have district/regional hospitals, and the population continue to be served by the existing health facilities

**Table 3:** Available maternity and delivery beds per 10,000 population

Facility	Population per district/region	Maternity beds		Delivery beds	
		N	Per 10,000	N	Per 10,000
A	2,479,461*	36	0.15	6	0.02
B	371,351*	2	0.05	2	0.05
C	199,592	29	1.45	5	0.25
D	123,854*	15	1.21	5	0.40
E	139,283*	12	0.86	3	0.22
F	112,331	4	0.36	1	0.09
G	141,584	13	0.92	2	0.14
H	108,816	16	1.47	4	0.37
I	65,706*	14	2.13	2	0.30
J	111,259	22	1.98	2	0.18
Total	3,853,237	163	0.42	32	0.08

*Population data provided from the 2010 Census report (Ghana Statistical Service [GSS], 2010)

* Further demarcation of regional and district boundaries was done between 2008 and 2019, adding two new regions from the original Northern region and ten new districts within the original Northern region. However, these newly created districts/regions do not have district/regional hospitals, and the population continues to be served by the existing health facilities

**Table 4:**
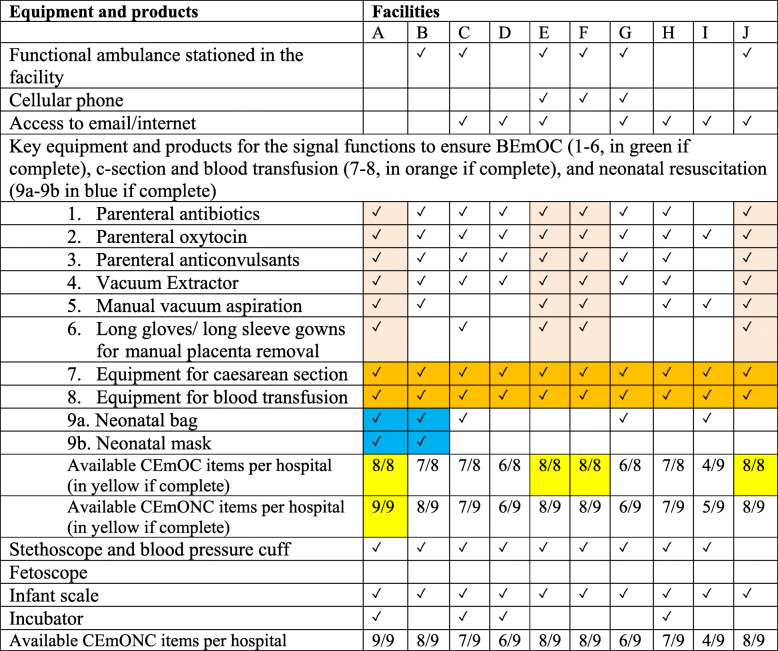
Availability of equipment and products for maternal healthcare, including referral

CEmONC (comprehensive emergency obstetric and newborn care)

### Ethical approval

The study protocol was assessed, approved, and ratified by the Navrongo Health Research Centre of the Ghana Health Service [NHRCIRB347]; and the Human Research Ethics Review Committee of the University of Technology Sydney, Australia [ETH19–4201]. Each participant provided written consent to participate in the study and to publish the data in a form that does not identify them in any way. All hospitals were de-identified and assigned identification letters.

## Results

### Births and ANC attendance

A total of 22,271 ANC visits from women to these hospitals occurred in the ten hospitals in the past 3 months preceding the study; however, 6072 birth events (cases) occurred within the same period. The number of ANC visits within the period ranged from 467 (facility E) to 7184 (facility A), with a ratio per 100,000 ranging between 289.74 (facility A) and 1858.08 (facility B). For the whole region, there were 577.98 ANC visits per 100,000 population. The number of births ranged from 161 (facility I) to 1400 (facility B) with a ratio per 100,000 ranging between 45.01 (facility A) and 608.83 (facility E). For the entire region, there were 157.58 births per 100,000 as indicated in Table 1.

### Maternal healthcare staff at the hospitals

There were 280 healthcare providers designated for maternity care within the region and ranged from no specialists and no university-trained nurses to 149 midwives. Across facilities, this ranged from 0 specialists/university-trained nurses (in all facilities) to 67 midwives in facility B. For the whole region, healthcare provider per 10,000 population ranged from 0 healthcare provider (specialists/university-trained nurses) per 10,000 population to 0.39 healthcare providers per 10,000 population (midwives).

### Maternity beds and partographs

The number of maternity beds per facility ranged from 2 (facility B) to 36 (facility A) with a ratio per 10,000 ranging between 0.05 (facility B) and 2.13 (facility I). For the whole region, there were a total of 163 beds with a ratio of 0.42 per 10,000. The number of maternity beds per facility ranged from 1 (facility F) to 6 (facility A) with a ratio per 10,000 ranging between 0.02 (facility A) and 0.40 (facility D). For the entire region, there were a total of 32 beds with a ratio of 0.08 per 10,000. All the ten hospitals reported that they used the partograph to monitor labour. However, six use partograph for all cases while four use partograph for some selected cases or conditions (data not shown).

### Availability of CEmONC and equipment for maternal healthcare including referral

Six hospitals had functional ambulances stationed on their premises, three (E, F, G) had cellular phones and seven were having access to email/internet. Four out of the ten hospitals had signal functions for BEmONC (A, E, F, J). However, only hospital A had all the signal functions for CEmONC. Hospital I had the least signal functions for CEmONC (i.e. four). All the hospitals had stethoscope, blood pressure cuffs and fetoscope except hospital J. Infant scale was available in all the hospitals; however, only four (A, C, D, H) had incubators.

### Guidelines/standards for the delivery, emergency care and referral

The respondents in all ten hospitals reported that they had guidelines or standards for delivery/emergency obstetric care. We sighted the guidelines in eight hospitals. Eight hospitals were using the Integrated Management of Pregnancy and Childbirth (IMPAC) guidelines and the National Guidelines for CEmOC. One hospital had a national guideline called Safe Motherhood protocol.

Five of the hospitals had guidelines for maternal referrals. Of these five hospitals, we sighted guidelines in two hospitals that had been adapted from the national referral policy and guidelines developed to fit their local context. These locally developed guidelines considered the distance and time spent when referring women to the regional hospital, which happens to be the destination of most referral cases. All the hospitals indicated that they have hospital referral forms; however, we saw these in nine hospitals.

Key informants of all ten hospitals reported that referral forms accompany all cases to the receiving hospital. Nine of the hospitals reported that they keep copies of the completed referred forms. When we assessed the individual items on the referral forms against the ones outlined by Ghana’s National Referral Policy and Guidelines, the ‘urgency of referral condition’ was not included on the referral forms used by hospitals A and I.

## Discussion

This study used the functionalist perspective to assess the capacity for maternal healthcare provision and maternal referral in the Northern region of Ghana. We found that human resources for maternal healthcare were very low compared with the WHO recommended ratio of doctors, nurses and midwives per 10,000 population [15], while some health staff categories were not available for maternity care. For instance, the hospitals with the highest ratio of doctors (0.30 per 10,000 population), nurses (0.46 per 10,000 population) and midwives (1.76 per 10,000 population) were far below the recommended threshold of 23 per 10, 000. None of the ten hospitals had a university-educated nurse, a specialist doctor, or allied health professionals such as social workers or physiotherapists working within maternal healthcare.

According to the Ghana Health Service and WHO standards, the ten hospitals included in the study are CEmONC level facilities with each serving a population between 75,000 and 250,000 [33]. According to the staffing norms of the Ministry of Health, a regional hospital should have at least 40 midwives [37], yet the regional hospital assessed in our study had only 19 midwives. Six of the district hospitals, also fell below the national level required minimum threshold of 10–84 [37]. The northern region, therefore, has a “critical shortage” of maternity health workers because according to WHO, a “critical shortage” of health workers occurs when less than 23 midwives, nurses and doctors serve a population of 10,000 [15, 16]. This reflects vast gaps between current guidelines and can compromise hospitals’ ability to function [29, 30, 38]. Thus, the shortage can have serious consequences for maternal mortality and morbidity; if more women tend up for care, the health providers may not be able to provide prompt service and the delay can lead to death thereby compromising the functionality of health facilities [3, 29, 30]. Similarly, the critical shortage of human resource for healthcare has been reported from other sub-Saharan African countries such as Mali, Uganda, Sudan and Nigeria [39, 40].

New initiatives need to be instituted to attract and retain maternity care staff especially nurses and midwives to the Northern Region as previous financial interventions such as allowances and accommodation schemes have not attracted adequate numbers [21]. Healthcare trainees from the Northern region may be recruited within the region to reduce the chances of non-indigenous healthcare providers returning to their home regions. Improving the working environment of staff may also be required for these incentives to be successful.

Additionally, ensuring a comfortable living environment of the healthcare providers and their immediate families (e.g. well-ventilated housing with adequate drainage that is close to extended family and quality schooling for children, as well as employment opportunities for spouses) can motivate health workers to remain in the region and provide quality care. A systematic review of maternal health interventions in resource-constrained countries found that interventions aimed at creating a supportive working environment such as revamping existing health facility infrastructure, enhancing drugs supply, consumables and equipment for obstetric care resulted in a 40 and 55% reduction in maternal mortality ratio and case fatality rate respectively [41].

Our study identified gaps in essential equipment and commodities, communication, and transportation for maternity healthcare. Only one hospital had complete CEmONC facilities while only four had facilities for complete CEmOC. Thus, there was a lack of essential equipment needed for triage and care management as specified by WHO, especially for neonatal resuscitation [36, 42]. The inadequacy of essential equipment and commodities for obstetric services has also been reported from Nigeria and Tanzania and could adversely affect healthcare delivery [29, 30, 43, 44]. Greater investment in the equipment for CEmONC is required to ensure the hospitals’ optimal functioning to improve maternal and newborn health within the region.

Hospital I had the highest maternity bed ratio (2.13 per 10,000 population). In comparison, the highest delivery bed ratio of 0.40 per 10,000 population was recorded in hospital D. According to the Ghana Health Service, a standard bed capacity for a district hospital in Ghana ranges between 60 and 120 with at least 11 beds designated for maternity ward [45]. Similarly, a regional hospital should have 150 to 200 beds [46]. Compared with these indicators, two district hospitals and the regional hospital fall short of the required bed capacity. This shortage can have adverse implications on the functionality of these health facilities [29, 30]. A longitudinal study from China indicated that the number of hospital beds per 1000 population is a significant predictor of maternal mortality [47]. Concerted efforts by the government to invest in maternity beds and other essential supplies are vital to improve care and prevent maternal mortality [48, 49].

Our study found that only the regional hospital and five district hospitals had a functional ambulance stationed at their premises. This implies that a greater proportion of women may have transportation challenges during referral and may experience delays in reaching the next facility [3]. This is consistent with evidence from Ethiopia where only 17% of surveyed facilities had dedicated functional ambulances [50]. A facility assessment in Tanzania also revealed that health facilities were less likely to have emergency transportation/ambulance system [10]. The importance of readily available and functional ambulances to improve maternal and newborn health cannot be overemphasised. In India, an average ambulance service intensity (i.e. 0.16 ambulances per million people) resulted in 0.75% (7.5 per 1000) and 1.1% (11 per 1000) probability of reduction in neonatal and infant deaths respectively [51]. This is an indication that reliable ambulance service is critical for healthcare facilities to function optimally [29, 30]. Novel approaches to transporting women to facilities particularly in emergencies are needed. These may include motorized or non-motorized commercial vehicles or vehicles by relatives using private transportation [52].

Only one of the ten hospitals (E) included in our study had a cellular phone available to facilitate maternal referrals. This may suggest the possibility of an inter-facility communication gap during referrals; a situation that does not favour a positive referral outcome (29, 30]. Inter-facility communication is critical for receiving facilities to prepare for the arrival of the woman [53]. The National Referral Policy and Guidelines of Ghana stress that whenever possible, communication with the receiving facility should be made. In addition, all referrals are expected to be accompanied by documentation that conforms to national and internationally acceptable standards [32, 42, 54]. Toll-free communication facilities within hospitals in countries such as in Bangladesh, Uganda, Rwanda has been found to enhance the inter-facility communication required for effective maternal referral [29, 30, 55–57].

Ghana’s National Referral Policy and Guidelines state that copies should be available in all units or departments of all hospitals nationwide [54]. Half of the surveyed hospitals in our study reported that they had referral guidelines and only two had readily available copies of the National Referral Policy and Guidelines. While all hospitals reported that they have guidelines or standards for the delivery of emergency obstetric care, copies could only be verified in eight hospitals. This may impact the quality of care and the monitoring of performance to ensure quality improvement. Ensuring the consistent application of guidelines through on-going monitoring may require capacity-building and mobilisation of maternal healthcare providers. The Advance in Labour and Risk Management (ALARM) Program is a well-known initiative that has contributed substantially to improving maternal healthcare quality in several low and middle-income countries through the enhanced monitoring of guideline compliance [58, 59].

The ALARM International Program, developed by the Society of Obstetricians and Gynaecologists of Canada, provides integrated clinically-oriented and evidence-based outreach visits and facility-based maternal death reviews. Program interventions include staff training in obstetric best practice and monthly audit cycles conducted according to WHO guidelines. Greater Criterion-based clinical audit (CBCA) scores were observed in intervention hospitals (68.2%) relative to control hospitals (64.5%) [59]. All these essential inputs can be guaranteed when there is adequate policy-driven healthcare financing by the central government [38] to improve policy management. The implementation of the ALARM program would considerably improve the monitoring of quality care in the Northern Region.

### Strengths and limitations

The study has some limitations. The functionalist theory that informed the study does not account for the adverse implications of social disorder, nor did it account for initiatives to improve the status quo. Thus, the theory does not acknowledge uncertainties such as conflicts or mayhem and their implications on healthcare provision and access. The theory is also limited as it does not propose ways to enhance or improve the current health system. This study was carried out in public hospitals and does not include any information about private facilities. However, public hospitals are the key service providers in the Northern Region as they deliver free maternal healthcare under the National Health insurance scheme. The availability of signal functions and some equipment were not observed but ascertained through interviews. Health facilities that provide BEmONC services such as Community-based Health Planning and Services (CHPS), clinics, polyclinics were also not included since the study’s focus was on CEmONC. Among the strengths of this study are the fact that it assessed all CEmONC services in the Northern region. Two authors with enormous survey administration and maternity healthcare delivery experience in the Ghanaian context gathered the data. Moreover, reported figures were verified from existing records to ensure accuracy.

## Conclusion

This study investigated hospitals’ readiness to deliver maternal care and refer women in the Northern region of Ghana. To our knowledge, this is the first study to assess maternal services in the region. We identified a number of issues indicating the need for considerable investment to address communication gaps and shortages of essential equipment to improve the efficiency, work safety, and motivation of the maternal health workforce in the region. Comprehensive health systems strengthening is needed at all levels requiring the implementation of current standards for hospitals and strategies such as performance-based financing to enhance the provision of maternal health services in the Northern Region.

## Supplementary Information

**Additional file 1.**

## Data Availability

The datasets used and/or analysed during the current study are available from the corresponding author on reasonable request.

## Abbreviations

ALARM: Advance in Labour and Risk Management
ANC: Antenatal Care
BEmONC: Basic Emergency Obstetric and Newborn Care
CBCA: Criterion-based clinical audit
CEmONC: Basic Emergency Obstetric and Newborn Care
CHPS: Community-based Health Planning and Services
EmOC: Emergency Obstetric Care
IMPAC: Integrated Management of Pregnancy and Childbirth
MMR: Maternal Mortality Ratio
SDG: Sustainable Development Goal
SBM-R: Standards-Based Management and Recognition
SPA: Service Provision Assessment
WHO: World Health Organisation
