# Supplementary material for: An assessment of hospital maternal health services in northern Ghana: a cross-sectional survey
Source: BMC Health Serv Res. 2020 Nov 26;20:1088. doi: 10.1186/s12913-020-05937-5 (PMC7690070; doi:10.1186/s12913-020-05937-5)
Supplement: Supplementary file 1 — Additional file 1. [file 12913_2020_5937_MOESM1_ESM.docx]

**Participant Information and Consent Form for Healthcare Providers**

My name is Edward Ameyaw, a PhD student in Public Health at the University of Technology Sydney. This study forms part of the requirements for the programme of study. You are invited to participate in this study. The study seeks to understand maternal referral practices in the Northern region. I would like to find out how maternal referral cases are managed in your hospital. The study does not assess your clinical expertise and how you carry out your duties. The findings of this study will provide information to develop better ways of improving maternal referrals and improving maternal and newborn health.

Please note that your participation in this study is entirely voluntary and you can choose not to answer any questions that you are uncomfortable with. You can also stop the interview at any moment without any risk. Any information you provide me will be strictly confidential and I will not disclose your identity to any third party without your express consent. Your participation in this study has some risks. You may experience psychological distress by recalling traumatic experience, however, I will refer you to a counselling unit if this occurs. This study will last for approximately 50 minutes. Please sign here if you are ready to participate in this study:

………………………………

Sign

Thank you for granting this interview out of your busy schedule.

**Facility Assessment Questionnaire**

Date_________________

Start Time_________________ End Time_________________

|  | **Section A: Respondent’s Information** | |
| --- | --- | --- |
| 1 | Position of respondent | ……………………………………………………………………………… |
| 2 | Sex of respondent | Male……………...………………..1  Female……………...….…………..2 |
| 3 | Years of experience in current position | 3-7 years……………....…….….….1  4-8 years……………….....………..2  9-13 years…………..….…………..3  14-18 years..………...….………….4  19 years and above..……………….5 |
| 4 | Years of working with current hospital | 3-7 years.….…………………….…1  4-8 years….………………………..2  9-13 years..…………………………3  14-18 years...……………..………...4  19 years and above….……………...5 |
|  | **Section B: Hospital Information** | |
| 5 | Name of Hospital | ………………………………………………………………….................................... |
| 6 | Status of Hospital | District Hospital....……….………...1  Regional Hospital……………..........2 |
| 7 | District Name | Tamale Metro.....…………………...1  Kumbungu District……….…...........2  Kpandai District………….………....3  Nanumba North Municipal……...….4  Zabzugu District…………………….5  Yendi Municipal…………………….6  Savelugu Municipal…………………7  Gushegu Municipal……………...….8  Saboba District……………...………9 |
| 8 | Location of hospital/town | ……………………………………  …………………………………… |
|  | **Section C: Referral system** | |
| 9 | Can you tell me about referral process at this hospital? | …………………………………………………………………………………………………………………………………………………………………………………… |
| 10 | Can you please give me an example of a typical case you recently referred? | ……………………………………………………………………………………………………………....................................... |
| 11 | How often do you receive feedback on referred cases? | Not at all…………………..…...…....1  Rarely……………………..…………2  Sometimes…………………..…….....3  Always..…………………….….……4 |
| 12 | What are the main challenges with referring women at your facility? Select all that apply | Transportation challenges……….….1  Low compliance from women.……..2  Inadequate human resource limiting referral accompaniment.....................3  Low human resource expertise……..4  Other, (specify)……………...………  ……………………………………………....................................................................5 |
| 13 | What improvements would you make to the referral processes at this hospital if you were able to? | ……………………………………………………………………………………………………………………………………….  ………………………………………………………………………………………… |
| 14 | Is the hospital having any referral guidelines for maternity and newborn cases? | Yes………………………….………1  No……………………………….….2 |
| 15 | If yes to Q14, can I see a copy of the referral guidelines your hospital uses? | Seen…………….…………………..1  Not seen……….…......…………….2 |
| 16 | Type of guideline | National referral policy and guidelines………………….……….1  WHO referral recommendations…...2  Other, (specify)………………….…..  ………………………………………3 |
|  | **Section D: International standards for the management of emergency obstetrics** | |
| 17 | Does the department have any guideline(s)/standard(s) for delivery care/emergency care? | Yes……………..……………………1  No…..……………………………….2 |
| 18 | If yes to Q17, can I see the guidelines/standards? | Seen……………………..…………..1  Not seen………….........…………….2 |
| 19 | Specific guidelines/standards | Integrated Management of  Pregnancy and Childbirth (IMPAC)…1  National Guidelines for comprehensive emergency  obstetric care (CEmOC)…………….2  Other, (specify)………………………  ………………………………………3 |
|  | **Section E: Human resource for health** | |
| 20 | How many women were delivered at this facility in the past three months? | ………………… |
| 21 | How many women sought antenatal care from this facility during the past three months? | ………………… |
| 22 | How many of these staff categories cater for maternal care services? |  |
|  | 1. General medical doctors | ………………… |
|  | 1. Specialist medical doctors (including Anesthesiologists & Pathologists) | ………………… |
|  | 1. Non-Physician Clinicians/Paramedical Professionals (including Clinical Officers, Medical Assistants, e.t.c.) | ………………… |
|  | 1. Anesthetists | ………………… |
|  | 1. Nursing professionals (excluding associate degree nurses) | ………………… |
|  | 1. Degree nurses | ………………… |
|  | 1. Midwifery professionals (excluding degree midwives) | ………………… |
|  | 1. Degree midwives | ………………… |
|  | 1. Enrolled nurse/enrolled midwife | ………………… |
|  | 1. Pharmacists/Pharmacy technologists/Pharmacy technicians/assistants | ………………… |
|  | 1. Laboratory scientists/technologists/technicians/assistants | ………………… |
|  | **Section F: Maternity information systems** | |
| 23 | Does the department you use referral form? | Yes……………..……1  No…………………….2 Que 28 |
| 24 | Can I see a copy of your referral form? | Seen………………………………...1  Not seen………......………………...2 |
| 25 | Look out if form has sections for the following: |  |
|  | 1. Name of woman | Present Absent |
|  | 1. Age/Date of Birth | Present Absent |
|  | 1. Health insurance status | Present Absent |
|  | 1. Address of woman | Present Absent |
|  | 1. Clinical history and examination findings | Present Absent |
|  | 1. Results of relevant investigations | Present Absent |
|  | 1. Diagnosis and treatment given | Present Absent |
|  | 1. Name, address and telephone number of referring facility | Present Absent |
|  | 1. Name, address and telephone number of facility being referred to | Present Absent |
|  | 1. Date of referral | Present Absent |
|  | 1. Time of referral | Present Absent |
|  | 1. Presence of the name of referring practitioner/clinician, signature and stamp | Present Absent |
|  | 1. Urgency of referral | Present Absent |
|  | 1. Reason for referral | Present Absent |
| 26 | Does the facility keep a copy of all filled referral forms when referring women? | Yes………………………………….1  No…………………………………..2 |
| 27 | Does filled referral form accompany all referred cases? | Yes………………………………….1  No…………………………………..2 |
|  | **Section G: Medicines, equipment and signal functions** | |
| 28 | Do providers of delivery services use partograph to monitor labour and delivery? | Yes.....................................................1  No……..……………………………2 |
| 29 | If yes to Q28, are partographs used for all cases or selectively (only for some cases)? | All cases…………………..………..1  Selectively…….…………..………..2 |
| 30 | How many dedicated maternity beds are available in this hospital? | None………………………………..1  1-19…………………………………2  20-39…………….………………….3  40-59………………….…………….4  60-79……………………….……….5  80-99……………………….….…….6  100-19………………….……….......7  120-139……………………………..8  140-159……………………………..9  160-179…………………………….10  180-199…………………………….11  200 and above……...………………12 |
| 31 | How many dedicated delivery beds are available in this hospital? | None………………………………..1  1-19…………………………………2  20-39…………….………………….3  40-59………………….…………….4  60-79……………………….……….5  80-99………………………….…….6  100-19………………….……….......7  120-139……………………………..8  140-159……………………………..9  160-179…………………………….10  180-199…………………………….11  200 and above……...………………12 |
| 32 | Does this facility have a functional ambulance or other vehicle for emergency transportation for maternity that is stationed at this facility and that operates from this facility? | Yes……………………………….....1  No…………………………………..2 |
| 33 | Does this facility have landline telephone/cellular telephone/private cellular phone to call outside to facilitate referral service? | Yes………………………………….1  No.…………………………………..2 |
| 34 | Is there access to email or internet via computer and/or mobile phone within the facility? | Yes………………………………….1  No…………………………………..2 |
| 35 | Do you have the following for maternity services: |  |
|  | 1. Incubator | Yes………………………………….1  No…………………………………..2 |
|  | 1. Manual vacuum extractor | Yes………………………………….1  No…………………………………..2 |
|  | 1. Infant scale | Yes…………………………….……1  No…………………………………..2 |
|  | 1. Stethoscope | Yes………………………………….1  No…………………………………..2 |
|  | 1. Fetal stethoscope | Yes………………………………….1  No…………………………………..2 |

**Thank you!!!**
